# Effects of induced electric field on the sensitivity of a two-compartment neuron model

**DOI:** 10.1371/journal.pone.0324523

**Published:** 2025-05-20

**Authors:** Chunhua Yuan, Rupei Chen, Xiangyu Li

**Affiliations:** School of Automation and Electrical Engineering, Shenyang Ligong University, Shenyang, China; Lanzhou University of Technology, CHINA

## Abstract

Sensitivity is one of the key characteristics of neurons in response to external stimuli. This study is based on a two-compartment Pinsky-Rinzel neuron model, which has been modified under the influence of a direct current induced electric field(DC-IEF). The research explores how this neuron model encodes stimuli from the DC-IEF, aiming to assess its sensitivity to firing in response to the induced electric field. Based on the two-compartment structural characteristics of the PR model neuron, the influence parameters of the model are altered under specific direct current stimulation to identify the state bifurcation points of the neuron at different parameters. At these bifurcation points, a DC-IEF is applied, and a planar graph is constructed to illustrate the relationship among firing rate, influence parameters, and electric field intensity. Through the analysis of the obtained data, it was found that PR neurons exhibit firing sensitivity to the DC-IEF. Furthermore, different influence parameters significantly affect their sensitivity and firing state.

## 1. Introduction

Neuronal sensitivity, referring to the ability of neurons to perceive and respond to external stimuli (e.g., electric fields and chemical substances), is fundamental to neural information processing and transmission. This property not only governs the normal function of neural networks but is also closely linked to various neurological disorders. For example, in patients with neuronal ceroid lipofuscinosis (NCL), changes in neuronal sensitivity may contribute to misdiagnosis [1]. During epileptogenesis, histone deacetylases (HDACs) emerge as potential therapeutic targets through the regulation of neuronal sensitivity [2]. The aberrant distribution of brain metabolites (NAA, GABA, and Glx) in individuals with autism further highlights alterations in neuronal sensitivity to external signals [3]. Moreover, other studies have demonstrated the potential of diverse compounds and therapeutic strategies in modulating neuronal sensitivity [4–7].

External electric fields have been demonstrated to affect neuronal sensitivity, notably through high-definition transcranial direct current stimulation (HD-tDCS), which enhances sensory functions, including improved force perception accuracy and the modulation of cerebral hemodynamics [8]. Research has indicated that electromagnetic fields can markedly modulate neuronal firing patterns, enhance firing rates, and facilitate the differentiation of neural stem cells into neuronal phenotypes [9,10]. Clinically, the potential of non-invasive brain stimulation techniques for the modulation of neurological and psychiatric disorders has become increasingly evident [11].

Further investigations have demonstrated that neuronal responses to induced electric fields are modulated by synaptic types, with electrochemical synapses displaying more intricate dynamic behaviors under electromagnetic field stimulation compared to electrical and chemical synapses [12]. Research employing the FitzHugh-Nagumo model suggests that the interplay between electric fields and external currents can elicit chaotic dynamics under certain conditions, thereby influencing neural network synchronization and system stability [13,14]. Moreover, advancements in the application of control theory to neuronal regulation continue to emerge. For example, fixed-time integral super-twisting sliding mode controllers, integrated with optogenetics, have been applied to epilepsy control in the Pinsky-Rinzel model [15], whereas adaptive barrier function terminal sliding mode control has also shown promise in specific epilepsy interventions [16].

Although previous studies have demonstrated the influence of electric fields on neuronal firing patterns, synaptic connectivity, and dynamic characteristics, most of these studies rely on single-compartment neuron models or idealized assumptions, limiting their ability to accurately depict the response of complex neural structures to electric fields. Furthermore, investigations into the effects of induced electric fields on neuronal sensitivity remain scarce. Most existing studies emphasize the impact of chemical agents or other external factors on biological sensitivity, whereas the specific mechanisms governing neuronal sensitivity under IEF conditions remain inadequately examined. To bridge this gap, this study utilizes the Pinsky-Rinzel (PR) model to comprehensively examine the regulatory influence of DC-IEF on PR neuronal firing patterns and sensitivity. By adopting a modeling approach that more closely reflects physiological conditions, this work aims to elucidate the role of IEF in neural regulation.

Building on the aforementioned background, this study hypothesizes that PR neurons exhibit pronounced sensitivity to DC-induced electric fields (DC-IEF), with their firing behavior and sensitivity being significantly regulated by neuronal model parameters, particularly coupling conductance and the potassium channel reversal potential. To test this hypothesis, this study systematically examines the impact of DC-IEF on neuronal firing properties within the PR model and incorporates the mean firing rate FFF as a quantitative indicator of neuronal firing characteristics. This study emphasizes the regulatory roles of coupling conductance and the potassium channel reversal potential in modulating neuronal firing rates and sensitivity. Furthermore, it investigates the firing rate–voltage amplitude ($F-V_{e}$ ) curve and three-dimensional vector plots ($F-g_{c}-V_{e}$ and $F-V_{k}-V_{e}$) to uncover the mechanisms by which induced electric fields exert their effects.

The innovation of this study lies in the development of an improved two-compartment neuron model under the influence of a direct current-induced electric field (DC-IEF), designed to more realistically simulate neuronal responses to external electric fields. Using this model, the study thoroughly investigates the sensitivity of Pinsky-Rinzel (PR) neurons to DC-IEF, with a particular focus on analyzing the regulatory effects of coupling conductance and potassium channel reversal potential on neuronal sensitivity. Additionally, the study further examines how these parameters influence neuronal firing characteristics, such as firing rate and firing patterns. Through these investigations, this study provides new theoretical insights into neuronal firing behavior under the influence of DC-IEF and offers potential physiological and pathological implications for research on neuron-originated diseases.

## 2. Materials and methods

### 2.1. The PR neuron model

**Fig 1:**
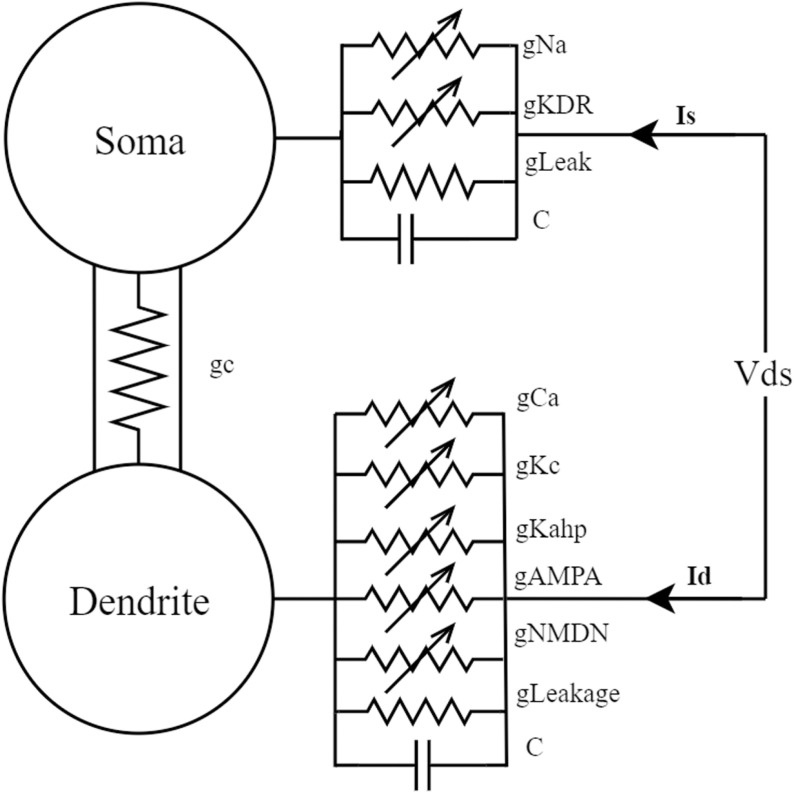
Simplified cable model of the two-compartment neuron in the CA3 region of the hippocampus.

The PR model [17] is a classical two-compartment neuronal model designed to describe the complex electrical activity of neurons in the hippocampus, particularly the pyramidal cells in the CA3 region. This model proposed by Pinsky and Rinzel in 1994, based on the fundamental principles of ion channels in the Hodgkin-Huxley model [18], and represents the neuron as two interconnected compartments: the dendritic compartment and the somatic compartment. Its structure is illustrated in Fig 1. This spatial extension allows for a more accurate simulation of the electrophysiological properties of different regions of the neuron. This is of significant importance for understanding how neurons integrate input signals from the dendrites and generate action potentials in the soma.

The interconnection between the dendritic compartment and the somatic compartment is a crucial component of the PR model. This connection simulates the authentic signal transmission process within the neuron, allowing the model to capture the dynamics of dendritic input reception and somatic output generation. The signal transmission between the two compartments may be influenced by various factors, among which the two most significant parameters are the coupling conductance ($g_{c}$) and the potassium channel reversal potential ($V_{k}$), both of which can be adjusted and analyzed within the model.


$$\begin{array}{cc} & C_{m}{\dot{V}}_{s}=-I_{sLeak}(V_{\text{s}})-I_{Na}(V_{\text{s}},h)-I_{KDR}(V_{\text{s}},n)+\frac{g_{c}(V_{d}-V_{s})}{p}+\frac{I_{s}}{p}\\ & C_{m}{\dot{V}}_{d}=-I_{dLeak}(V_{\text{d}})-I_{Ca}(V_{\text{d}},s)-I_{KAHP}(V_{\text{d}},q)-I_{kc}(V_{\text{d}},Ca,c)+\frac{g_{c}(V_{s}-V_{d})}{1-p}+\frac{I_{d}}{1-p} \end{array}$$ (1)

Among these,


$$\begin{array}{cc} & {I_{sLea}}_{k}=g_{L}\cdot (V_{S}-V_{L})\\ & I_{Na}=g_{Na}\cdot m_{\infty }^{2}\cdot h\cdot (V_{S}-V_{Na})\\ & I_{KDR}=g_{KDR}n(V_{s}-v_{k})\\ & I_{DS}^{in}=g_{C}\cdot (V_{d}-V_{S})\\ & I_{dLeak}=g_{L}\cdot (V_{d}-V_{L})\\ & I_{ca}=g_{ca}s^{2}(V_{d}-V_{ca})\\ & \chi (Ca)=\min(Ca/250,1)\\ & I_{KC}=g_{KC}c\chi (Ca)(V_{d}-V_{k})\\ & I_{KAHP}=g_{KAHP}q(V_{d}-V_{K}) \end{array}$$ (2)

The kinetic characteristics of the gating variables for each ion channel are described by the following equations:


$$\frac{dx}{dt}=\frac{x_{\infty }(V)-x}{\tau_{x}(V)}$$ (3)

Specifically, $x_{\infty }=\frac{\alpha_{x}}{\left(\alpha_{x}+\beta_{x}\right)}$ and $\tau_{x}=\frac{1}{\left(\alpha_{x}+\beta_{x}\right)}$, where (x=m, h, n, s, c, q),α andβ represent the corresponding rate functions (see Table 1 for details).

**Table 1:** Rate Functions of the Gating Variables for Each Ion Channel.

	Forward (α)	Backward (β)
**Variable m**	$\alpha_{m}=\frac{0.32(-13.1-V_{s})}{\exp((-13.1-V_{s})/4)-1}$	$\beta_{m}=\frac{0.28(V_{s}-40.1)}{\exp((V_{s}-40.1)/5)-1}$
**Variable h**	$\alpha_{h}=0.128\exp(\frac{17.0-V_{s}}{18.0})$	$\beta_{h}=\frac{4}{1+\exp((-40.0-V_{s})/5)}$
**Variable n**	$\alpha_{n}=\frac{0.016(35.1-V_{s})}{\exp((35.1-V_{s})/5)-1}$	$\beta_{n}=0.25\exp(0.5-0.025V_{s})$
**Variable S**	$\alpha_{s}=\frac{1.6}{1+\exp(-0.072(V_{d}-65))}$	$\beta_{s}=\frac{0.02(V_{d}-51.1)}{\exp((V_{d}-51.1)/5)-1}$
**Variable C**	$\alpha_{c}=\frac{\exp((V_{d}-10.0)/11-(V_{d}-6.5)/27)}{18.975}$ $\alpha_{c}=2\exp(\frac{65-V_{d}}{27})$	$\beta_{c}=2\exp(\frac{6.5-V_{d}}{27})-\alpha_{c}\;V_{d}\leq 50.0$ $\beta_{c}=0\;V_{d}>50.0$
**Variable q**	$\alpha_{q}=\min(0.00002Ca,0.01)$	$\beta_{q}=0.001\;$

The dynamic equation for calcium ion concentration is as follows:


$$\dot{C}a=-0.13I_{Ca}-0.075Ca$$ (4)

In this study, the fourth-order Runge-Kutta (RK4) method is utilized to numerically solve the differential equations governing the Pinsky-Rinzel neuronal model. The RK4 method determines the evolution of neuronal state variables through a four-step predictor-corrector scheme, providing high computational accuracy. The detailed computational procedure is outlined as follows:


$$\begin{array}{cc} & k_{1}=\Delta t\cdot f(x_{n})\\ & k_{2}=\Delta t\cdot f(x_{n}+\frac{k_{1}}{2})\\ & k_{3}=\Delta t\cdot f(x_{n}+\frac{k_{2}}{2})\\ & k_{4}=\Delta t\cdot f(x_{n}+k_{3})\\ & x_{n+1}=x_{n}+\frac{1}{6}k_{1}+\frac{1}{3}k_{2}+\frac{1}{3}k_{3}+\frac{1}{6}k_{4} \end{array}$$ (5)

In the equation, $x_{n+1}$ denotes the state variable at step n+1; f(x) is determined by the differential equations of the Pinsky-Rinzel model. The time step is specified as Δt=0.1ms. The total simulation duration is specified as T=7000ms, and the total number of iterations is given by N=T/Δt=70000. The initial parameter values are given as follows:$V_{s}=8.22594127701169$, $V_{d}=11.2873513664516$, h=0.657103951268693, n=0.0575840069166615, s=0.0586561971436294, c=0.0328693668351334, q=0.461747452058436, ca=46.9558464653944.

The values of the remaining variable parameters are presented in Table 2.

**Table 2:** Parameter Values for the Pinsky-Rinzel Model.

	Values
**The Membrane Area of the Soma Region,** ρ **(Proportional Area)**	0.5
**Total Membrane Area,** $\begin{array}{c} Area \end{array}$	6 × 10^–6^(*cm*^2^)(Somatic Radius: 5*µm*)
**Coupling Conductance Between the Two Compartments,** $\begin{array}{c} g_{c} \end{array}$	$1,\;2.1,\;5,\;10,\;15,\;25(mS/cm^{2})$
**Membrane Capacitance,** $\begin{array}{c} C_{m} \end{array}$	$3.0\mu F/cm^{2}$
**Reversal Potential of Sodium Ions,** $\begin{array}{c} V_{Na} \end{array}$	120 (mV)
**Reversal Potential of Calcium Ions,** $\begin{array}{c} V_{Ca} \end{array}$	140 (mV)
**Reversal Potential of Potassium Ions,** $\begin{array}{c} V_{K} \end{array}$	−15(mV)
**Leak current potential,** $\begin{array}{c} V_{L} \end{array}$	0 (*mV*)
**Synaptic Potential,** $\begin{array}{c} V_{syn} \end{array}$	60 (*mV*)
**leak conductance,** $\begin{array}{c} g_{L} \end{array}$	$0.1\;(mS/cm^{2})$
**Sodium Conductance,** $\begin{array}{c} g_{Na} \end{array}$	$30\;(mS/cm^{2})$
**Delayed Rectifier Potassium Conductance,** $\begin{array}{c} g_{KDR} \end{array}$	$15\;(mS/cm^{2})$
**Calcium Conductance,** $\begin{array}{c} g_{Ca} \end{array}$	$2.1\;(mS/cm^{2})$)
**Potassium Afterhyperpolarization Conductance,** $\begin{array}{c} g_{KAHP} \end{array}$	$0.8\;(mS/cm^{2})$
**Potassium Conductance,** $\begin{array}{c} g_{KC} \end{array}$	$15\;(mS/cm^{2})$
**Somatic Current,** $\begin{array}{c} I_{S} \end{array}$	$0(\mu A/cm^{2})$
**Dendritic Current,** $\begin{array}{c} I_{D} \end{array}$	$0.7(\mu A/cm^{2})$

### 2.2. An improved PR neuron model under the influence of a DC-IEF

According to reference [19–21], When an induced electric field is applied, the depolarization voltage  Δ V across the neuronal membrane satisfies:


$$\Delta \dot{V}+\frac{\Delta V}{\tau }=\frac{\lambda E}{\tau }$$ (6)

In the equation, τ denotes the time constant (typically 10^−10^ seconds), representing the rate of charge accumulation on the cell membrane; λ is the polarization length of the neuron [22,23]; E denotes the applied electric field. When a DC-induced electric field with amplitudeA is applied to the neuron, E(t)=A, the depolarization voltage  Δ v(t) across the membrane satisfies:


Δv(t)=λE(t)=λA (7)

Considering the depolarization ΔV of the cell membrane as an external perturbation applied [24] to the membrane potential V(t), the PR model is then modified from equation (1) to:


$$\begin{array}{cc} & C_{m}\frac{d(V_{s}+\Delta V)}{dt}=-I_{sLeak}(V_{\text{s}}+\Delta V)-I_{Na}(V_{\text{s}}+\Delta V,h)-I_{KDR}(V_{\text{s}}+\Delta V,n)\\ & \;+\frac{g_{c}(V_{d}-V_{s})}{p}+\frac{I_{s}}{p}\\ & C_{m}\frac{d(V_{d}+\Delta V)}{dt}=-I_{dLeak}(V_{\text{d}}+\Delta V)-I_{Ca}(V_{\text{d}}+\Delta V,s)-I_{KAHP}(V_{\text{d}}+\Delta V,q)\\ & \;-I_{kc}(V_{\text{d}}+\Delta V,Ca,c)+\frac{g_{c}(V_{s}-V_{d})}{1-p}+\frac{I_{d}}{1-p} \end{array}$$ (8)

The induced electric field in this study is a DC-IEF, which takes the following form:


$$\;V_{e}=A$$ (9)

In this equation, A represents the amplitude voltage of the DC-IEF. This study assumes that the induced electric field is equivalent to the depolarization voltage ΔV caused on the cell membrane, i.e., $\;\;\;\Delta \;\;\;V=V_{e}$, therefore, the variable $I_{e}$ induced by the DC-induced electric field on the left-hand side of the two equations in Equation 8 is:


$$I_{e}=C_{\text{m}}\frac{d(\Delta V)}{dt}=0$$ (10)

Therefore, under the effect of the DC-induced electric field, the electric field amplitude $V_{e}=A$, and the current variable $I_{e}=0$.

In summary, the improved Pinsky-Rinzel neuronal model incorporating the induced electric field is as follows:


$$\begin{array}{cc} & C_{m}{\dot{V}}_{s}=-I_{sLeak}(V_{\text{s}}+V_{e})-I_{Na}(V_{\text{s}}+V_{e},h)-I_{KDR}(V_{\text{s}}+V_{e},n)\\ & \;+\frac{g_{c}(V_{\text{s}}-V_{d})}{p}+\frac{I_{s}}{p}\\ & C_{m}{\dot{V}}_{d}=-I_{dLeak}(V_{\text{d}}+V_{e})-I_{Ca}(V_{\text{d}}+V_{e},s)-I_{KAHP}(V_{\text{d}}+V_{e},q)\\ & \;-I_{kc}(V_{\text{d}}+V_{e},Ca,c)+\frac{g_{c}(V_{\text{s}}-V_{d})}{1-p}+\frac{I_{d}}{1-p} \end{array}$$ (11)

The expressions for other variables and the values of other parameters are in accordance with the standard PR model.

### 2.3. Analysis of the firing activity of PR neurons under different influencing parameters

The coupling conductance ($g_{c}$) plays a crucial role in regulating the strength of synaptic transmission and neuronal interactions. Variations in $g_{c}$ influence not only neuronal responsiveness to external stimuli but also its functional performance across various physiological states. Furthermore, the extracellular potassium ion concentration critically regulates the reversal potential of potassium channels ($V_{k}$), and excessively high potassium levels may trigger various neurological disorders.

Thus, variations in $g_{c}$ and $V_{k}$ are essential for understanding neuronal dynamics. This study considers these two parameters as critical factors in examining the firing characteristics of PR neurons under different DC-IEF conditions, and analyze the sensitivity of neuronal firing to DC-IEF.

**Fig 2:**
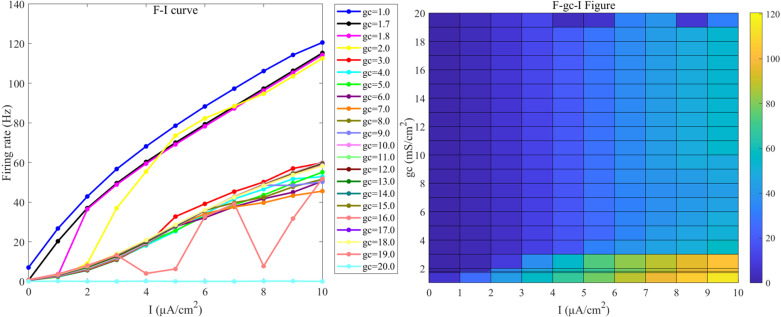
The F−I curve of PR neurons under direct current input (left) and the $\begin{array}{c} F-g_{c}-I \end{array}$ plane plot (right). The model parameter is set to *V*_*k*_ = -15 mV. In the F−I curve, the differently colored curves represent the firing frequencies of neurons at various coupling conductance ($g_{c}$) values, specifically $g_{c}$ values of [1.0,1.7,1.8] mS/cm² and [2,20] mS/cm² (with an interval of 1 mS/cm²); In the $F-\;g_{c}-I$ plane plot, the x-axis represents the input current I, ranging from 0 to 10 *µA/cm*^*2*^, with intervals of 1 *µA/cm*^*2*^, that is, from no DC stimulation to strong DC stimulation. The y-axis represents the coupling conductance ($g_{c}$), where different colors in the plot correspond to different firing rates (*F*).

**Fig 3:**
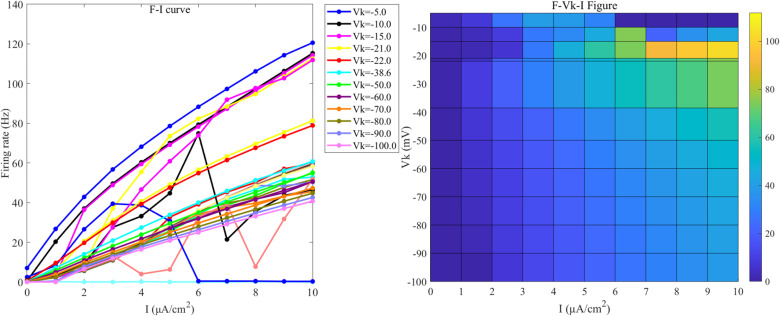
The F−I curve of PR neurons under direct current input (left) and the $\begin{array}{c} F-V_{k}-I \end{array}$ plane plot (right). The model parameter is set to *g*_*c*_ = 2.1 mS/cm². In the F−I curve, the differently colored curves represent the firing frequencies of neurons at different reversal potentials ($V_{k}$) of potassium ions, specifically $V_{k}$ values of [-5,-10,-15,-21,-22,-38.56,-50,-60,-7-,-80,-90,-100]mV; In the $F-V_{k}-I$ plane plot, the x-axis represents the input current I, ranging from 0 to 10 *µA/cm*^*2*^, with intervals of 1 *µA/cm*^*2*^. The y-axis represents the reversal potential ($V_{k}$) of potassium ions, where different colors in the plot correspond to different firin*g* rates (F).

Without additional direct current stimulation (as illustrated in Figs 2 and 3), the firing frequency of neurons with varying parameter values during the simulation remains low, making it difficult to investigate changes in firing frequency and patterns under external electric field.

When the synaptic current slightly increases, causing $I_{d}$ to rise to 1 μA/cm², the neuron transitions into a firing state. Therefore, to investigate the effect of external electric fields on neuronal sensitivity, a DC stimulus must be applied first. In this study, a weak DC stimulus (*I*_*s*_ = 0μA/cm², *I*_*d*_ = 1μA/cm²) is used to sustain the neuron’s baseline firing frequency.

**Fig 4:**
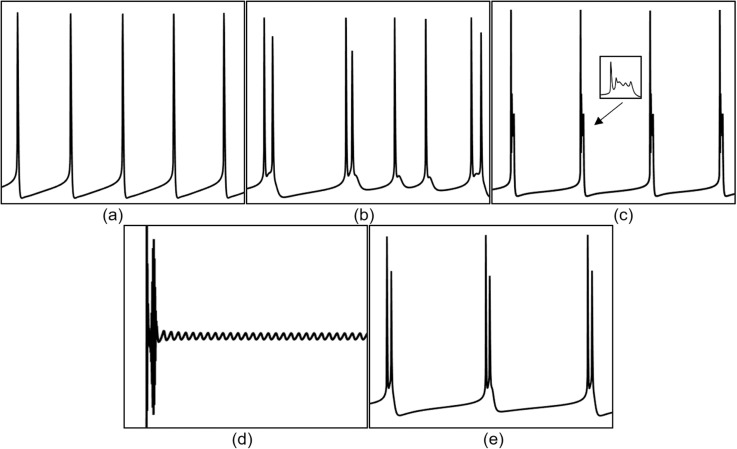
Different Firing States of PR Neurons. (a) periodic spike firing state, (b) burst-spike alternating firing state, (c) periodic burst firing state, (d) subthreshold oscillatory state, (e) period-2 burst firing state.

The PR model exhibits diverse firing patterns under different coupling conductance ($g_{c}$) and reversal potential ($V_{k}$) of potassium channels. The following analyzes bifurcation states under different $g_{c}$ and $V_{k}$ conditions with weak direct current stimulation: For different $g_{c}$ values, $V_{k}$ is set to -15 mV (at this value, neurons exhibit more detailed firing characteristics). When $g_{c}$ is greater than 0 and less than 1.7 mS/cm², the neurons exhibit a periodic spike firing state (as illustrated in Fig 4a); When $g_{c}$ is 1.8 mS/cm², the neurons demonstrate a burst-spike alternating firing state (as illustrated in Fig 4b); When $g_{c}$ is greater than or equal to 1.8 and less than 10 mS/cm², the neurons transition into a periodic burst firing state (as illustrated in Fig 4c); When $g_{c}$ is greater than or equal to 10 mS/cm² and less than 20 mS/cm², the firing state of the neurons reverts to a periodic spike firing state; When $g_{c}$ is greater than or equal to 20 mS/cm², the neurons stop firing.

Under varying $V_{k}$ conditions, with $g_{c}$ set to 2.1 mS/cm²(the standard value), the neurons display a subthreshold oscillatory state (as illustrated in Fig 4d) when $V_{k}$ exceeds -5 mV; When $V_{k}$ is less than -5 mV and greater than -22 mV, the neurons enter a periodic burst firing state, where, at an external electric field amplitude of -21 mV, the neurons display period-2 burst firing state (as illustrated in Fig 4e); When $V_{k}$ is less than or equal to -22 mV and greater than -91 mV, the neurons transition into a periodic spike firing state; When $V_{k}$ exceeds -91 mV, the neurons revert to a resting state.

Since, under weak DC stimulation, the neuron’s firing frequency drops to 0 when $g_{c}$ exceeds 20 mS/cm² (as shown by the curve for *g*_*c*_ = 20 in Fig 2), and since bifurcation occurs at 1.7 and 1.8 mS/cm², therefore, in this study, when investigating the sensitivity of neurons to the induced electric field under different gc values, the gc is varied from {1.0, 1.7, 1.8} mS/cm² and from [2,20] mS/cm² (with a step size of 1 mS/cm²), Particular attention is given to the firing state and sensitivity of neurons at gc values of 1, 1.7, 1.8, and 10 mS/cm².

When exploring the sensitivity of neurons to the induced electric field under varying $V_{k}$ values, since the neuron’s firing frequency drops to 0 when $V_{k}$ is below -100 mV under weak DC stimulation, bifurcation occurs at -5, -21, and -22 mV, and -38.56 mV is the value under standard conditions, therefore, the $V_{k}$ values are {-5, -10, -15, -21, -22, -38.56} mV and [-50, -100] mV in increments of 10 mV, with a focus on investigating the firing state and sensitivity of neurons at $V_{k}$ values of -5, -21, and -22 mV.

## 3. Results

### 3.1. Effects of *g*_*c*_ on the firing sensitivity of neurons under a DC-IEF

**Fig 5:**
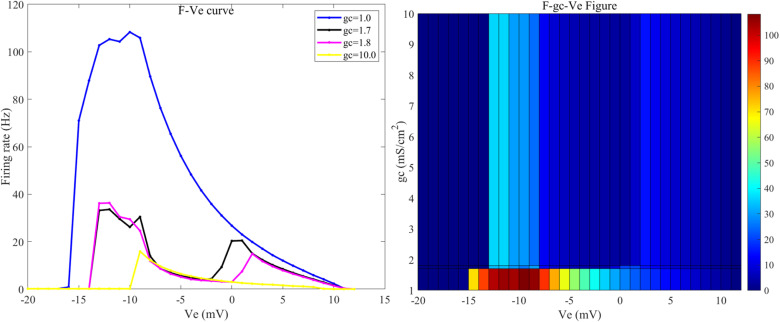
The $\begin{array}{c} F-V_{e} \end{array}$ curves of PR neurons with varying coupling conductance ($g_{c}$) under static direct current electric field (left) and the 2D mapping of $\begin{array}{c} F-g_{c}-V_{e} \end{array}$ (right). Where $g_{c}$ values are set to 1, 1.7, 1.8, and 10 mS/cm², and the external electric field ($V_{e}$) ranges from -20 mV to 12 mV, with intervals of 1 mV.

**Fig 6:**
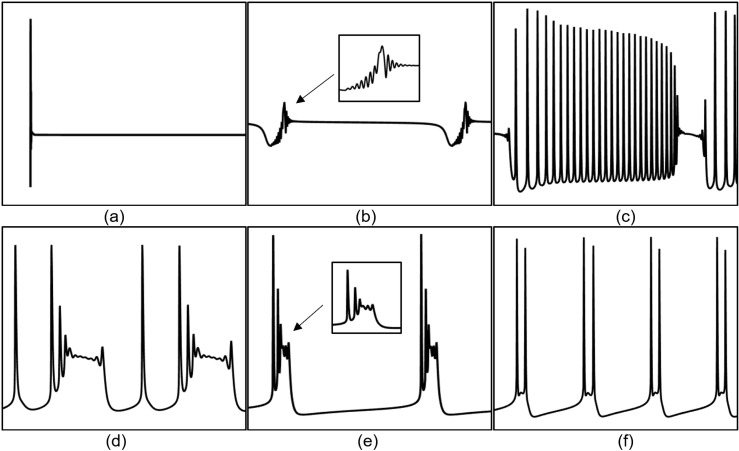
Different firing states of PR neurons under the induced electric field. (a) depolarization block state, (b) biphasic oscillation state, (c) fast bursting state, (d) mixed-mode oscillatory firing state, (e) periodic burst firing state, (f) two-cycle bursting state accompanied by subthreshold oscillations.

When the Coupling conductance ($g_{c}$ value) is 1 mS/cm² (blue line in Fig 5), when the neuron is subjected to an induced electric field $V_{e}$ below -17 mV, it enters a depolarization block state (as shown in Fig 6a, where the membrane potential remains at a high level for a prolonged period, preventing the generation of new action potentials), and when $V_{e}$ is 17 mV, the neuron exhibits biphasic-oscillations state (as shown in Fig 6b). In both these states, the neuron does not exhibit firing, and it is not sensitive to the external electric field. When the amplitude of the external electric field ($V_{e}$) is within the range of [-16, -10] mV, the neurons display a fast bursting state (as shown in Fig 6c). As the amplitude of the external electric field continues to increase, when $V_{e}$ falls within the range of [-9, 11) mV, the neurons fully transition to a spike firing state; when $V_{e}$ exceeds 11 mV, the neurons revert to a resting state. Thus, at this conductance level, the sensitivity range of neurons to the induced electric field is [-16, 11] mV, and other ranges do not show sensitivity.

When the $g_{c}$ value is 1.7 mS/cm² (black line in Fig 5), when the induced electric field $V_{e}$ is below -18 mV, the neuron enters a depolarization block state, and when $V_{e}$ is in the range of [-18, -13] mV, the neuron enters a biphasic-oscillation state.When $V_{e}$ exceeds 11 mV, the neurons revert to a resting state. Therefore, the sensitivity range of neurons to the applied induced electric field is [-13, 11] mV. Within this range, when $V_{e}$ falls within the interval [-13, -9] mV, the neurons exhibit a mixed-mode oscillatory firing state (as shown in Fig 6d); when $V_{e}$ is in the range of [-8, -2] mV, the neurons transition to a periodic burst firing state (as shown in Fig 6e); when $V_{e}$ is in the range of [-1, 0] mV, the neurons return to a cluster-spike alternating firing state; when $V_{e}$ is 1 mV, the neurons display a two-cycle bursting state accompanied by subthreshold oscillations (as shown in Fig 6f); and when $V_{e}$ falls within the range of [2,11) mV, the neurons enter a periodic spike firing state.

When the $g_{c}$ value increases to 1.8 mS/cm² (magenta line in Fig 5), the firing state of the neurons under the external electric field resembles that at 1.7 mS/cm², with a sensitivity range to the induced electric field of [-14, 11] mV. Within this range, when $V_{e}$ falls within the interval [-13, -9] mV, the neurons display a mixed-mode oscillatory firing state; when $V_{e}$ is in the range of [-8, 1) mV, the neurons transition to a periodic burst firing state; when $V_{e}$ is 2 mV, the neurons display a two-cycle bursting state accompanied by subthreshold oscillations; and when $V_{e}$ falls within the range of [3,10] mV, the neurons enter a periodic spike firing state.

When the $g_{c}$ value is increased to 10 mS/cm² (yellow line in Fig 5, the sensitivity range of the neurons to the induced electric field is [-10, 9] mV. Within this range, when $V_{e}$ falls within the interval [-9, -7] mV, the neurons display a periodic burst firing state; when $V_{e}$ is in the range of [-6, 9) mV, the neurons transition to a periodic spike firing state; when $V_{e}$ exceeds 9 mV, the neurons revert to a resting state; and when $V_{e}$ falls below -10 mV, the neurons enter a depolarization block state.

**Fig 7:**
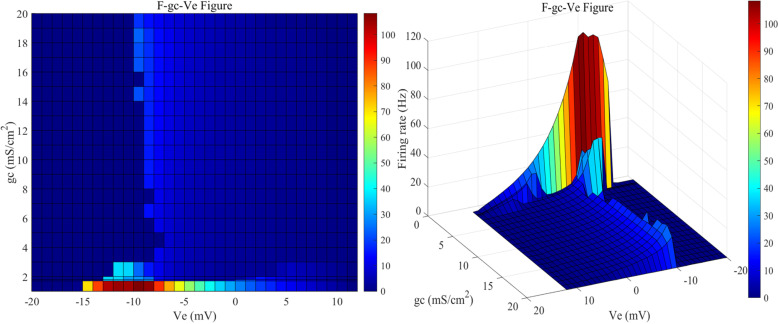
The 2D mapping of $\begin{array}{c} F-g_{c}-V_{e} \end{array}$ for PR neurons with varying coupling conductance ($g_{c}$) under static direct current electric field (left) and the 3D plot (right). Where $g_{c}$ values are set to 1, 1.7, 1.8, and range from 2 to 20 mS/cm² (with intervals of 1 mS/cm²), and the external electric field ($V_{e}$) ranges from -20 mV to 12 mV, with intervals of 1 mV.

As illustrated in Fig 7, when the $g_{c}$ value is 2 mS/cm², the sensitivity range of the neurons to the applied direct current induced electric field is [-13, 11] mV, and beyond this range, the neurons are insensitive to the direct current electric field. Within this range, the firing activity of the neurons varies similarly to that at 1.8 mS/cm². When the $g_{c}$ value is between 3 and 20 mS/cm², the firing frequency and firing pattern of the neurons under the influence of the external electric field remain relatively stable, with a sensitivity range of [-10, 10] mV. Within this range, $g_{c}$ has little effect on the firing state and firing frequency of the neuron after the application of an external electric field. The changes in firing frequency follow the following pattern: as the intensity of the forward electric field increases, the firing frequency gradually decreases until it stops; conversely, under an enhanced reverse electric field, the firing frequency gradually increases, and after reaching a certain frequency, the neuron enters a depolarization block state.

In summary, when $g_{c}$ is small, the electrical activity of the soma and dendrites is more independent, leading to a more significant potential gradient between them under the effect of DC-IEF, thus increasing the neuron’s sensitivity to the external electric field. As $g_{c}$ increases, the coupling of electrical signals between the soma and dendrites strengthens, and the spatial distribution of the membrane potential becomes more balanced, effectively dissipating the local potential difference induced by DC-IEF, thus decreasing the neuron’s overall sensitivity to the electric field.

### 3.2. Effects of *V*_*k*_ on the firing sensitivity of neurons under a DC-IEF

**Fig 8:**
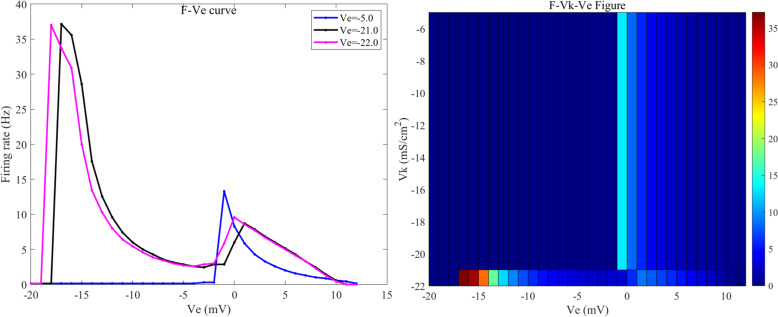
The $\begin{array}{c} F-V_{e} \end{array}$ curves of the potassium ion channels in PR neurons at different reversal potentials ($\begin{array}{c} V_{k} \end{array}$) under static direct current electric field (left) and the 2D mapping of $\begin{array}{c} F-V_{k}-V_{e} \end{array}$ (right). Where $\begin{array}{c} V_{k} \end{array}$ values are set to -1, -2, and -3 mV, and the external electric field ($\begin{array}{c} V_{e} \end{array}$) ranges from -50 mV to 50 mV, with intervals of 1 mV.

When the $V_{k}$ value is -5 mV (blue line in Fig 8, the sensitivity range of the neurons to the external electric field is [-1, 12] mV. Within this electric field range, the neurons display a periodic burst firing state. When the applied induced electric field $V_{e}$ is less than -11 mV, the neuron enters a depolarization block state. When $V_{e}$ is in the range of [-11, -1] mV, the neuron exhibits subthreshold oscillatory state; and when $V_{e}$ exceeds 12 mV, the neurons revert to a resting state.

When the $V_{k}$ value is -21 mV (black line in Fig 8), when the applied induced electric field $V_{e}$ is less than -22 mV, the neuron enters a depolarization block state. When $V_{e}$ is in the range of [-22, -18] mV, the neuron exhibits subthreshold oscillatory state;when $V_{e}$ falls within the interval [-17, -13] mV, the neurons display a mixed-mode oscillatory firing state; when $V_{e}$ is in the range of [-12, 0] mV, the neurons transition to a periodic burst firing state; when $V_{e}$ falls within the interval [1,10] mV, the neurons display a periodic spike firing state; and when $V_{e}$ exceeds 11 mV, the neurons revert to a resting state. Thus, the sensitivity range under the external electric field is [-17, 11] mV.

When the $V_{k}$ value increases to -22 mV (magenta line in Fig 8), the firing state and pattern of the neurons remain consistent with those at a k value of -21 mV, although the intervals differ slightly. In this scenario, when $V_{e}$ is less than -23 mV, the neuron enters a depolarization block state. When $V_{e}$ is in the range of [-23,-19] mV, the neuron exhibits subthreshold oscillatory state; when $V_{e}$ falls within the interval [-18, -16] mV, the neurons display a cluster-spike alternating firing state; when $V_{e}$ is in the range of [-15, -1] mV, the neurons transition to a periodic burst firing state; when $V_{e}$ falls within the interval [0, 10] mV, the neurons display a periodic spike firing state; and when $V_{e}$ exceeds 11 mV, the neurons revert to a resting state. Thus, under these parameters, the sensitivity range of the neurons to the external electric field is [-18, 11] mV.

**Fig 9:**
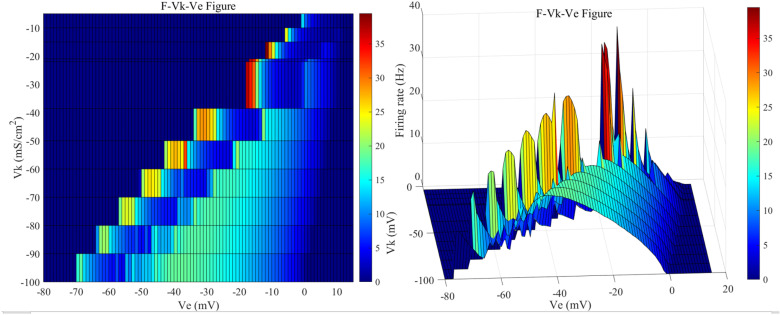
The 2D mapping of $\begin{array}{c} F-V_{k}-V_{e} \end{array}$ for the reversal potential ($\begin{array}{c} V_{k} \end{array}$) of potassium ion channels in PR neurons under static direct current electric field (left) and the 3D plot (right). Where $\begin{array}{c} V_{k} \end{array}$ values are set to {-5,-10,-15,-21,-22,-38.56}mV和[-50, -100]mV (with a step size of 10 mV), and the external electric field ($\begin{array}{c} V_{e} \end{array}$) ranges from -50 mV to 50 mV, with intervals of 1 mV.

The firing frequencies of neurons in the presence of the applied induced electric field at various $V_{k}$ values are illustrated in Fig 9. When $V_{k}$ is -38.56 mV, the sensitivity range of the neurons to the direct current electric field is [-35, 9] mV; when $V_{k}$ is -50 mV, the sensitivity range is [-44, 7] mV; when $V_{k}$ is -60 mV, the range is [-52, 6] mV; when $V_{k}$ is -70 mV, the range is [-58, 4] mV; when $V_{k}$ is -80 mV, the range is [-65, 2] mV; when $V_{k}$ is -90 mV, the range is [-65, 1] mV; and when $V_{k}$ is -100 mV, the range is [-71, -1] mV. As the $V_{k}$ value decreases progressively, the sensitivity range of the neurons under the external electric field keeps expanding.

When $V_{k}$ falls below -40 mV, the trend of changes in the firing state of the neurons under the external electric field remains generally similar, but the maximum firing frequency gradually decreases. As $V_{k}$ decreases, the amplitude of the applied induced electric field corresponding to the maximum firing frequency of the neurons also gradually diminishes. When $V_{k}$ is within the range of [-5, -15] mV, the maximum firing frequency of neurons increases as the absolute value of the reversal potential $V_{k}$ increases; the applied direct current induced electric field corresponding to the maximum firing frequency also gradually decreases.

The magnitude of $V_{k}$ is determined by the intracellular (${\left[K^{+}\right]}_{i}$) and extracellular (${\left[K^{+}\right]}_{o}$) potassium ion concentrations. A smaller absolute value of $V_{k}$ generally suggests that ${\left[K^{+}\right]}_{i}$ is significantly higher than ${\left[K^{+}\right]}_{o}$, bringing the neuronal membrane potential closer to a hyperpolarized state, enhancing potassium efflux, stabilizing the membrane potential, and suppressing DC-IEF-induced excitability changes. Conversely, a larger absolute value of $V_{k}$ indicates that ${\left[K^{+}\right]}_{o}$ is relatively high, bringing the membrane potential closer to a depolarized state. This reduction in the driving force of potassium channels decreases potassium efflux, thereby enhancing the modulatory effect of DC-IEF on the neuronal membrane potential.

### 3.3. Pattern analysis of *g*_*c*_ and *V*_*k*_ influences on neuronal firing under DC-IEF

This study establishes a neuronal model influenced by direct current induced electric field based on the standard Pinsky-Rinzel neuron model, and analyzes the firing states under different conductance ($g_{c}$) and reversal potential ($V_{k}$) values during weak direct current stimulation. The study primarily investigates the effects of $g_{c}$ and $V_{k}$ values on the sensitivity and firing state changes of PR neurons in direct current induced electric field during bifurcation conditions. Based on the simulation analysis, the following principles are concluded:

➀ When *g*_*c*_= 10 mS/cm², the neuron exhibits a relatively high firing frequency, with a sensitivity range to the external electric field of [-16, 11] mV.

➁ As $g_{c}$ increases, the sensitivity range gradually decreases. When $g_{c}$ falls within the range of [3,20] mS/cm², the sensitivity range stabilizes at [-10, 10] mV. In this case, the firing state and frequency of the neuron remain nearly unchanged under different amplitudes of the external electric field.

➂ With an increase in the absolute value of $V_{k}$, the sensitivity range of the neuron to the applied induced electric field gradually expands. The range in the forward electric field decreases, while the range in the reverse DC-IEF increases. For example, the range expands from [-1, 12] mV at *V*_*k*_ = -5 mV to [-77, -1] mV at *V*_*k*_ = 100mV. However, the firing state of the neuron remains essentially unchanged under the external electric field.

➃ When $V_{k}$ is in the range of [−5, −15] mV, the maximum firing frequency of the neuron gradually increases as the absolute value of $V_{k}$ increases. However, when $V_{k}$ is less than −40 mV and greater than −100 mV, the maximum firing frequency gradually decreases.

➄ The external induced electric field amplitude corresponding to the maximum firing frequency decreases as $V_{k}$ decreases.

When the amplitude of the applied direct current induced electric field exceeds the sensitivity range, the neurons cease periodic firing and enter either a resting state or depolarization block state, resulting in a loss of sensitivity to the DC-IEF.

## 4. Discussion

### 4.1. Limitations

Although this study elucidates the effects of DC-IEF on the firing characteristics of the Pinsky-Rinzel two-compartment neuron model, some limitations still exist. First, while the Pinsky-Rinzel model employed in this study provides an accurate representation of pyramidal neuron properties in the thalamocortical circuit, it is still a simplified model that does not fully account for the entire physiological complexity of neurons. For instance, the model does not account for dynamic changes in the neuronal microenvironment, synaptic plasticity, or more intricate ion channel regulation mechanisms, potentially limiting the generalizability of the findings. Furthermore, this study focuses solely on two key parameters—coupling conductance and potassium channel reversal potential—while neglecting other physiological factors that could affect neuronal firing properties, such as calcium channel dynamics and neurotransmitter modulation. Additionally, this study examines only the effects of DC-IEF, without exploring AC electric fields or other forms of external stimulation, which may constrain the comprehension of broader mechanisms underlying electric field modulation. Therefore, future research could integrate more sophisticated neuron models, extend parameter analyses, and investigate diverse electric field types and other external stimuli to better understand their effects on neuronal dynamics.

### 4.2. Implications

Despite these limitations, the findings provide new insights into the understanding of neuron-originated diseases. Previous studies have demonstrated that aberrant neuronal firing patterns are pivotal in the onset and progression of epilepsy, Parkinson’s disease, and other neurological disorders [2,4,20]. External electric fields modulate neuronal excitability and firing patterns [25,26], thereby affecting the pathogenesis and potential therapeutic strategies of these diseases and playing a crucial role in neuromodulation [27]. Therefore, this study not only enhances the understanding of how induced electric fields modulate neuronal activity but also offers new theoretical foundations for the diagnosis and treatment of neurological disorders.

### 4.3. Future directions

Future studies can be further extended in the following directions:

(1) Incorporating more sophisticated neuronal models, such as multi-compartment models that integrate diverse synaptic types and network connectivity properties, to enhance the physiological relevance of the research.
(2) Conducting systematic investigations of parameter variations under different physiological and pathological conditions to validate the generalizability of the findings and elucidate the fine-tuned regulatory effects of electric fields on neuronal dynamics.
(3) Investigating other forms of external stimulation, including alternating current-induced electric fields (AC-IEF), chemical signaling, or mechanical stimuli, to further elucidate neuronal responses to environmental changes.
(4) Integrating experimental studies to validate model predictions and ensure alignment between theoretical outcomes and real physiological phenomena, thereby enhancing the translational value of the research.

By addressing the current study’s limitations and advancing future research, we can further elucidate the role of electric fields in neuromodulation, offering a stronger theoretical foundation for both fundamental research and clinical interventions in neurological disorders.

## 5. Conclusion

This study systematically explores the effects of DC-IEF on the firing properties of the PR two-compartment neuronal model and examines how two key parameters—coupling conductance and potassium reversal potential—regulate neuronal firing rate and sensitivity under induced electric fields. The results demonstrate that DC-IEF significantly influence neuronal firing patterns and excitability, while variations in coupling conductance and potassium reversal potential further refine this regulation, underscoring the crucial role of induced electric fields in neuromodulation.

## Supporting information

S1 File Supporting Information. This ZIP file contains all supplementary materials, including the raw data supporting the figures and tables presented in the article.
(ZIP)
