# Supplementary figures and images for: Effects of induced electric field on the sensitivity of a two-compartment neuron model
Source: PLoS One. 2025 May 20;20(5):e0324523. doi: 10.1371/journal.pone.0324523 (PMC12091897; doi:10.1371/journal.pone.0324523)

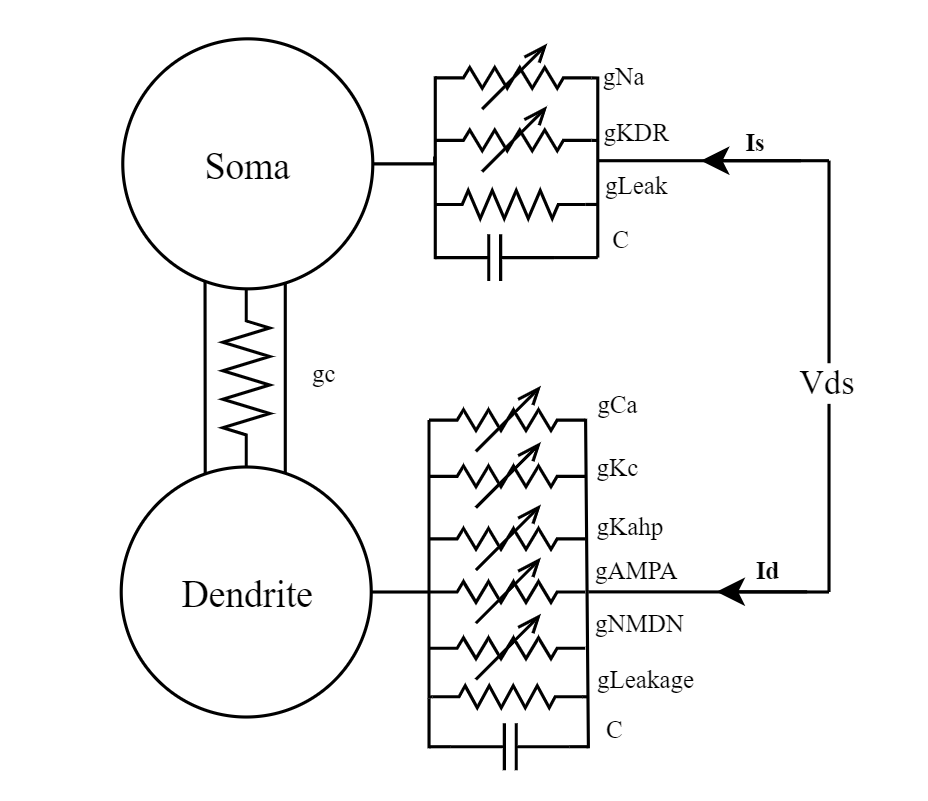

Supplement: S1 File — This ZIP file contains all supplementary materials, including the raw data supporting the figures and tables presented in the article. (ZIP) [file pone.0324523.s001.zip › S1_Fig.tif]
